# 554. Clinical Impact of Monoclonal Antibody Therapy with SARS-CoV-2 Infection

**DOI:** 10.1093/ofid/ofab466.752

**Published:** 2021-12-04

**Authors:** Inderjit Mann, Melinda Monteforte, Roderick Go

**Affiliations:** 1 Renaissance School of Medicine at Stony Brook University, Stonybrook, New York; 2 Stony Brook University Hospital, Stony Brook, NY

## Abstract

**Background:**

The novel coronavirus SARS-CoV2 is the causative agent for COVID-19 responsible for the ongoing global pandemic. The spike protein on its surface binds to the angiotensin-converting enzyme 2 receptor helps to enter human cells. Neutralizing antibodies to this protein can be protective and helpful in alleviating symptoms. Monoclonal antibodies (mAb) have been utilized in the U.S. under an emergency use authorization by the FDA, including bamlanivimab (BAM) and casirivimab-imdevimab (CAR/IMD). We report our experience of using COVID mAb.

**Methods:**

We conducted a retrospective chart review of patients that received CAR/IMD or BAM between December 1st, 2020, and May 15th, 2021. Medical records were reviewed to determine demographic and clinical information as well as tolerability and effectiveness of mAb.

**Results:**

463 patients with mild to moderate symptoms of SARS-CoV2 received mAb: 355(176 Men) BAM, 108(53 Men) CAR/IMD. The median BMI was 31 (17.4 to 62.5), 85% Caucasian, 4% Asian, 3% African American, 4% Hispanic, 4% others. The average duration of symptoms was 3.4 days and included cough (74%), malaise (71%), Headache (28%), dyspnea (28%), rhinorrhea (25%), fever (20%), diarrhea (18%), and anosmia (14%). Risk factors included hypertension (65%), diabetes mellitus (32%), coronary artery disease (22%), asthma (16%), COPD (9%), CHF (9%), CKD (8%), active malignancy (6%), and immunocompromised state (7%). Those who received BAM were older (p=0.000) and have underlying dementia and congestive heart failure (p=0.025 and 0.034, respectively). 27 patients (2 CAR/IMG, 25 BAM) got admitted to the hospital due to worsening of their respiratory status and were treated for COVID-19. 4 patients in the BAM group and 0 in the CAR/IMD group died. 2 patients developed a mild allergic reaction to CAR/IMD, no other side effects were reported in both groups. 37 patients (19 CAR/IMD, 18 BAM) received mRNA COVID vaccine prior. Overall mortality rate was 0.8%. There was no significant difference between BAM and CAR/IMR in terms of hospitalization (p= 0.104) or mortality (p=0.268).

**Conclusion:**

Treatment with BAM versus CAR/IMR was well tolerated and resulted in similar outcomes in terms of hospitalization or mortality.

**Disclosures:**

**All Authors**: No reported disclosures

